# Retraction Note: Molecular analysis of the effects of Piroxicam and Cisplatin on mesothelioma cells growth and viability

**DOI:** 10.1186/s12967-022-03834-5

**Published:** 2022-12-27

**Authors:** Alessandra Verdina, Irene Cardillo, Angela Nebbioso, Rossella Galati, Simona Menegozzo, Lucia Altucci, Ada Sacchi, Alfonso Baldi

**Affiliations:** 1 Laboratory D, Dept. for the Development of Therapeutic Programs, CRS, Regina Elena Cancer Institute, Via delle Messi d’Oro 156, 00158 Rome, Italy; 2 Department of General Pathology and Oncology, “Centro Sperimentale S. Andrea delle Dame”, Second University of Naples, Via Costantinopoli 16, 80138 Naples, Italy; 3 Department of Biochemistry and Biophysics, Section of Pathology, Second University of Naples, Via L. Armanni 5, 80138 Naples, Italy; 4 Campania Regional Operating Center (COR) of the National Mesothelioma Registry (ReNaM) and Department of Experimental Medicine, Second University of Naples, Via Costantinopoli 16, 80138 Naples, Italy

**Retraction: Journal of Translational Medicine 2008, 6:27** **https://doi.org/10.1186/1479-5876-6-27**

The Editor-in-Chief has retracted this article [1]. After publication, concerns were raised regarding potential issues in the western blot images presented in the figures. Specifically:

- Two pairs of bands appear highly similar in Fig. 5a—MSTO C CycD1 (CTRL 3 h and 6 h) and P + C Actin (CTRL 6 h and 24 h).
- A number of bands appear highly similar in Fig. 6b P + C Actin.
- Also in Fig. 6b, NCI P p21 bands (CTRL 6 h and 24 h) appear highly similar to P + C p21 bands (CTRL 3 h and 6 h).
- All NCI Actin bands appear to be highly similar in Figs. 5a and 6b.
- NCI C Actin bands in Figs. 5a and 6b appear to be highly similar but rotated 180 degrees, with the same bands representing different groups.
- In Fig. 6b NCI blots, some backgrounds appear inconsistent and contain unexpected breaks or changes between bands.

As the article was published in 2008, the authors are unable to provide the raw data used to produce these figures. The Editor-in-Chief therefore no longer has confidence in the presented data.

Alfonso Baldi does not agree to this retraction. Angela Nebbioso and Lucia Altucci have not explicitly stated whether they agree to this retraction. Simona Menegozzo has not responded to any correspondence from the editor or publisher about this retraction. The Publisher has not been able to obtain a current email address for author Alessandra Verdina, Irene Cardillo, Rossella Galati and Ada Sacchi.

